# Single Viruses on the Fluorescence Microscope: Imaging Molecular Mobility, Interactions and Structure Sheds New Light on Viral Replication

**DOI:** 10.3390/v10050250

**Published:** 2018-05-10

**Authors:** Nagma Parveen, Doortje Borrenberghs, Susana Rocha, Jelle Hendrix

**Affiliations:** 1 Laboratory for Photochemistry and Spectroscopy, Molecular Imaging and Photonics Division, Chemistry Department, KU Leuven, B-3001 Leuven, Belgium; nagma.parveen@kuleuven.be (N.P.); doortjeborrenberghs@gmail.com (D.B.); susana.rocha@kuleuven.be (S.R.); 2 Dynamic Bioimaging Lab, Advanced Optical Microscopy Centre and Biomedical Research Institute (BIOMED), Hasselt University, B-3590 Diepenbeek, Belgium

**Keywords:** wide-field fluorescence microscopy, confocal laser scanning microscopy, super-resolution microscopy, single virus imaging, raster image correlation spectroscopy, single particle tracking, Förster resonance energy transfer, HIV, simian virus 40, oligomerization, stoichiometry

## Abstract

Viruses are simple agents exhibiting complex reproductive mechanisms. Decades of research have provided crucial basic insights, antiviral medication and moderately successful gene therapy trials. The most infectious viral particle is, however, not always the most abundant one in a population, questioning the utility of classic ensemble-averaging virology. Indeed, viral replication is often not particularly efficient, prone to errors or containing parallel routes. Here, we review different single-molecule sensitive fluorescence methods that we employ routinely to investigate viruses. We provide a brief overview of the microscopy hardware needed and discuss the different methods and their application. In particular, we review how we applied (i) single-molecule Förster resonance energy transfer (smFRET) to probe the subviral human immunodeficiency virus (HIV-1) integrase (IN) quaternary structure; (ii) single particle tracking to study interactions of the simian virus 40 with membranes; (iii) 3D confocal microscopy and smFRET to quantify the HIV-1 pre-integration complex content and quaternary structure; (iv) image correlation spectroscopy to quantify the cytosolic HIV-1 Gag assembly, and finally; (v) super-resolution microscopy to characterize the interaction of HIV-1 with tetherin during assembly. We hope this review is an incentive for setting up and applying similar single-virus imaging studies in daily virology practice.

## 1. Introduction

Animal viruses are small (typically 20–400 nm in diameter) [1], genetically simple infectious biological entities that require a living host organism to replicate. Viruses invade cells of living organisms and hijack the cellular machinery for integration of their DNA/RNA into the host’s genome to produce their progeny. Often, however, viral cell entry erroneously does not lead to reproduction, which is in part related to their intrinsic primitive nature [2,3]. For example, structural/functional heterogeneity and instability among viruses in a population is typically large, pointing to error-prone viral biogenesis. Also, viruses can often take different routes for passing different steps in the viral lifecycle, for example cellular entry [4,5], suggesting steps are either redundant or error-prone. Viral enzymes such as the human immunodeficiency virus (HIV) reverse transcriptase lack proof-reading machinery, resulting in large genetic mutation rates [6]. Ensemble detection methods do not allow distinguishing functionally different viral sub-populations, as they provide an average readout and are naturally skewed towards the behavior of the majority of viruses. Additionally, it is possible that ensemble methods miss out on visualizing and quantifying minor yet possible infectious sub-populations. Therefore, this provides strong arguments for investigating viruses at the level of single viral entities, rather than at the ensemble level. Of course, when single-virus imaging would be performed, ensemble information is not lost.

Fluorescence microscopy is an ideal method to study viruses one at a time. Fluorescence is widely employed in the life sciences, as it specifically allows highlighting particular components in complex biological specimens in a minimally invasive manner. It can also be applied in multiple colors, in real-time and, with the proper hardware, even single fluorescent molecules can be visualized accurately. Important parameters for fluorescence imaging are spatial and temporal resolution. Although conventional fluorescence microscopes are diffraction-limited (lateral resolution of 200–350 nm) [7], the advent of super-resolution fluorescence microscopy techniques has made it possible to achieve lateral resolutions down to 20–30 nm [8,9,10]. In terms of temporal resolution, fluorescence microscopes allow the study of dynamical processes on the macroscopic timescale (seconds, minutes, hours) down to the picosecond timescale. The latter timescale can be used, for example, to sense the environment in close proximity to the fluorescing dye (pH, ionic strength, crowding, molecular interactions, etc.). Many types of fluorescence microscopes are commercially available, although many research groups nowadays assemble them *de novo* for specialized purposes. Finally, fluorescence imaging is not restricted to taking pretty images or recording videos: images and image series can also be analyzed via advanced mathematical algorithms to extract quantitative molecular information. For example, spatial image correlation can reveal biomolecular mobility, stoichiometry or interactions [11,12,13] and Förster resonance energy transfer analysis can be used to obtain information on protein–protein interactions or protein conformation [14,15,16].

In this review, we first provide a brief overview of the hardware that we routinely use for single-molecule sensitive imaging compatible with studying single viruses. Then, we discuss the different fluorescence imaging modalities and analysis algorithms that we have used in our virology experimentation to quantify biophysical parameters such as molecular mobility, interactions and quaternary structure. With each method, we briefly discuss the particular step or aspect in the viral replication cycle that it can be used to study and describe the advantage(s) of the method in virology experiments. Our hope is that with this review, virologists will see the potential of these methods, and apply them for unravelling other key aspects of viruses and viral replication.

## 2. Single Virus Imaging Hardware

Typically, a fluorescence microscope is built up of an excitation unit, a microscope body and a detection unit, all of which are enclosed in a protective box when part of a commercial instrument or home-built on an optical table. In the excitation unit, lasers of specific wavelength (typically 405, 440, 488, 514, 561 and 635 nm) are combined for the excitation of different fluorophores. Their coherent, temporally stable and monochromatic output ensures high-quality imaging. The lasers are combined using dichroic mirrors that transmit/reflect light depending on its wavelength. The combined beam is then guided to the microscope, possibly through an acousto-optical tunable filter (AOTF) to allow fast and easy tuning of the transmitted power at each wavelength. After entering the microscope, the excitation light is reflected towards the objective lens via a high-quality (>95% reflection/transmission, ultraflat) polychroic mirror. The objective lens subsequently guides the excitation light to the sample, and simultaneously collects the fluorescence light in the so-called epi-mode. For high-performance imaging, a 60× or 100× magnification objective with the highest possible light-collection efficiency, expressed as the numerical aperture (*NA*) (*NA* = 1.2 for a water-immersion objective; *NA* = 1.49 for an oil-immersion objective), is used to collect as much as possible of the emitted light, and to image the specimen at the highest possible spatial resolution. The lateral resolution is given by the Rayleigh criterion as the smallest distance (*d*) between two points that can still be resolved, or *d* = 0.61*λ*/*NA* (*λ* is the wavelength of the light used). The polychroic mirror subsequently transmits the emission light, which is then guided to the detection unit. If multiple fluorophores are imaged simultaneously, the emission light is first split into different spectral bands using high-quality dichroic mirrors. The resulting bands are cleaned up spectrally using emission filters that block nonsense light such as residual laser reflections, or scattered light, from reaching the detector (a reduction of >10^6^-fold), and that transmit the fluorophore’s emission with high efficiency (>95%). Finally, the light is digitized using an ultrasensitive (detection efficiency > 50%) detector.

Imaging single particles, complexes or viruses (containing one or more fluorophores) is possible if their fluorescence is significantly higher than the background. In general, when the so-called signal-to-noise ratio (SNR) is larger than 5, an imaging system is said to be single-molecule sensitive. To achieve this sensitivity, excellently performing fluorophores are typically used (organic dyes or fluorescent proteins with >50% fluorescence quantum yield and good photostability), and the microscope is typically equipped with the best possible excitation source (lasers rather than lamps), optics (lenses, mirrors, filters, etc.), objective lens and detectors. In our single-virus imaging studies, we have used three single-molecule sensitive fluorescence microscopy modalities: camera-based microscopy (Figure S1) and conventional confocal microscopy (Figure S2), as well as a more advanced confocal microscope using pulsed interleaved excitation that allows artifact-free multicolor imaging (Figure S3) [17,18,19,20,21]. The interested reader is referred to the Supporting Information for a detailed review of each modality.

## 3. Förster Resonance Energy Transfer Probes the Human Immunodeficiency Virus (HIV-1) Integrase Quaternary Structure

Integrase (IN) is the retroviral enzyme that trims the 3’-ends of the newly synthesized blunt-ended double stranded viral DNA (vDNA), and catalyzes vDNA insertion into the host genome [22,23]. Via cross-linking and western blotting [24] and high-resolution structural techniques [24,25,26,27,28,29,30,31], IN has been shown to functionally oligomerize throughout the viral replication cycle into dimeric [25,29,31], tetrameric [26,27,28] or even higher order oligomeric species [24,30]. Next, LEDGF/p75 is a human transcriptional coactivator that tethers the intasome, the functional IN-DNA complex, to chromatin, thereby facilitating and targeting viral integration. LEDGF/p75 also stabilizes IN tetramers by interacting with the IN dimer interface. Inhibitors of the IN-LEDGF/p75 interaction could potentially put IN in an altered, non-functional oligomerization state, yet no one has directly shown this. Therefore, we set out to develop an assay based on Förster resonance energy transfer (FRET) to test small molecules affecting the IN quaternary structure inside virions.

**Figure 1:**
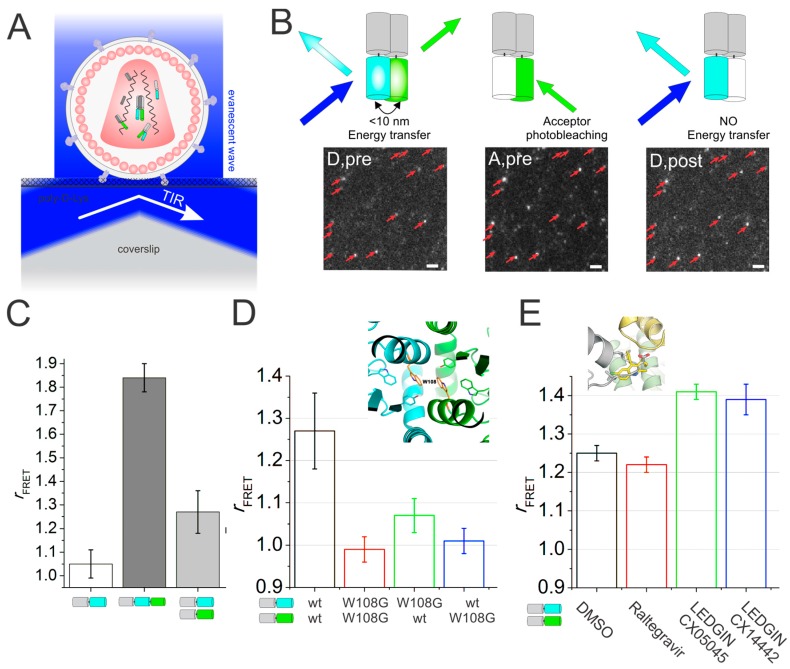
(**A**) Cartoon of total internal reflection fluorescence microscopy (TIRFM) imaging of mature human immunodeficiency virus (HIV-1) viral particles containing fluorescently labeled IN. Single-virus TIRFM enables clear imaging of immobilized single HIV viral particles in a small region (~200 nm) close to the coverslip at high signal-to-noise ratio (SNR). (**B**) The principle of acceptor photobleaching Förster resonance energy transfer (FRET) in HIV-1 viral particles. “Pre” and “post” describes the sample pre- and post-acceptor photobleaching. (left panel) The fluorescence of mTFP1 in viral particles containing donor and acceptor FP (Donor, pre) capable of FRET (<10 nm) is quenched by the proximal acceptor (mVenus). After photobleaching of the acceptor (middle panel), the donor is dequenched (right panel). Scale bar = 1 μm. Red arrows indicate fluorescent HIV-1 particles. (**C**) Mean FRET ratio of HIV_IN-mTFP1+IN-mVenus_ (light gray), HIV_IN-mTFP1_ (white) and HIV_IN-mTFP1-mVenus_ (dark grey). (**D**) (top) Crystal structure of the IN catalytic core dimer interface. W108G is indicated as orange sticks colored by atom. (bottom) mean FRET ratio of wild type (HIV_IN-mTFP1+IN-mVenus_) and mutant (IN^W108G^). (**E**) (top) Co-crystal structure of LEDGINs bound in the LEDGF/p75 binding pocket of HIV-IN (green and yellow). (bottom) Influence of adding compounds during viral production on the mean FRET ratio. ©Borrenberghs et al., 2014. Originally published in ACS NANO. https://pubs.acs.org/doi/pdf/10.1021/nn406615v [18].

FRET is the non-radiative energy transfer from an excited donor fluorophore to an acceptor fluorophore, provided their spectra overlap and their spacing is within 10 nm. The efficiency of the FRET process is 50% at the Förster distance (*R*_0_, about 5 nm for most FRET pairs [32]). Around *R*_0_, FRET is extremely sensitive to the inter-probe distance, rendering FRET (in contrast to e.g., classical or even super-resolution microscopy-based co-localization analysis) extraordinarily sensitive for probing protein–protein interactions and even protein structures (Table 1). Many methods to quantify FRET exist, relying solely on the donor intensity or fluorescence lifetime, on the acceptor intensity, or both on the donor and acceptor intensity. For our assay, we quantified FRET using only the donor intensity pre- and post-acceptor photobleaching (illustrated in Figure 1B), as this did not require any data correction a posteriori (e.g., spectral bleed-through or between-channel corrections). Finally, although FRET imaging can be performed with basically any microscopy modality, here we used total internal reflection fluorescence microscopy (TIRFM) [18] (reviewed in Figure S1), because of the high SNR, the large field of view (allowing the imaging of multiple viruses simultaneously) and the high power of continuous-wave lasers used for widefield microscopy (allowing efficient acceptor photobleaching). FRET studies have already provided fundamental insights to better understand viral replication, such as viral entry [33] or fusion [34,35], the activity of the reverse transcriptase enzyme [36,37], and viral assembly [16].

As FRET pair dyes, we used the fluorescent proteins (FPs) mTFP1 (donor) and mVenus (acceptor) [18]. At the time, this pair exhibited the highest percentage of FRETting molecules (because of the good protein folding and maturation), and the highest *R*_0_ value (6.1 nm) for FPs [38]. We chose FPs specifically and not, for example, organic dyes because of the straightforward stoichiometric labeling via genetic fusion. We did have to follow a special Vpr-trans-incorporation approach [39] to incorporate the FP-labeled IN into the viruses, as HIV-1 does not tolerate any large insertions in its genome downstream of the *pol* gene.

In practice, we targeted donor- and acceptor-labeled IN to assembling virions, purified the produced viruses, coated them on a microscopy coverslip and imaged them via TIRFM (Figure 1A). By imaging the FRET donor, bleaching the acceptor and imaging the donor again, we could then probe the IN quaternary structure inside virions (Figure 1B). Indeed, as compared to a negative or positive control, particles containing donor- and acceptor-labeling IN indeed exhibited a significant FRET signal (Figure 1C). Since a specific point mutation (W108G) [40] disrupted this FRET signal (Figure 1D), our data was indicative of a higher-order IN structure inside viruses. At the time, this was the first direct proof for IN oligomers inside HIV-1 virions. As a proof of principle that our assay could potentially be used to screen for enhancers of IN oligomerization, we tested different compounds: Raltegravir, the IN strand transfer inhibitor with no known effect on IN oligomerization, or LEDGINs, small molecule inhibitors of the IN-LEDGF/p75 interaction that are known to affect the oligomerization of recombinant IN in vitro [41,42,43]. Interestingly, for the latter the FRET signal was significantly larger (Figure 1E), directly proving that when LEDGINs are added during viral production, the IN quaternary structure is functionally altered. At the time, this was the first direct proof that LEDGIN compounds exhibited an additional effect to HIV-1 replication, besides the mere competitive inhibition of the IN-LEDGF/p75 interaction.

On a more general note, this work illustrated that viruses can be exploited as nanoscopic, miniaturized “test tubes” for probing, in principle, any protein-protein interaction via single-molecule FRET.

## 4. Single Particle Tracking to Study Simian Virus 40 Membrane Attachment

**Figure 2:**
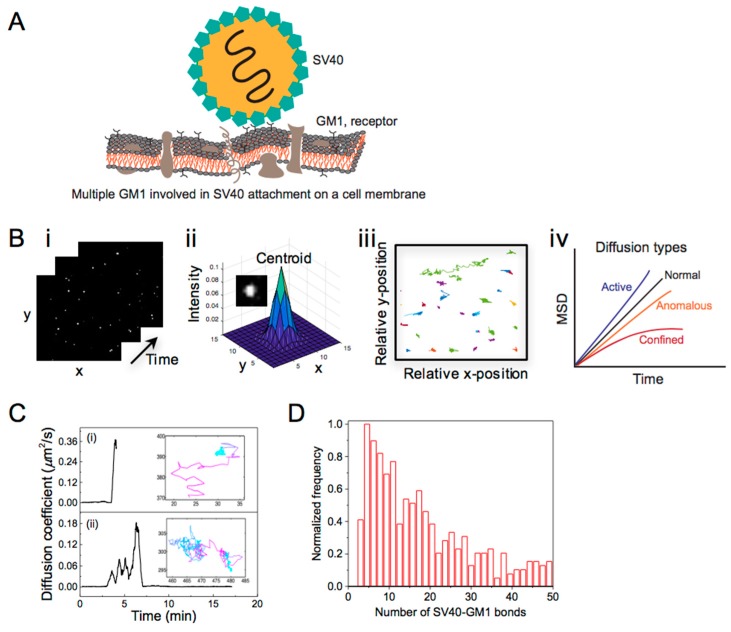
(**A**) Scheme of SV40 attached to the cell membrane via multiple copies of a glycosphingolipid receptor, GM1. The number of SV40-GM1 bonds involve in the virus attachment was determined with single particle tracking (SPT) analysis. (**B**) (**i**) Time-lapse images (25 × 25 μm^2^) of single viruses; (**ii**) fitting of the image of a single virus with a 2D Gaussian function; (**iii**) tracks of the detected viruses and (**iv**) mean square displacement (MSD) vs. time plot showing different diffusion types. (**C**) Time-resolved diffusion coefficient evaluated from the SPT analysis displaying transient mobility of SV40 particles during their lateral displacement on a supported phospholipid-bilayer with a few molar percent of GM1. The transient changes in the *D* of the particle over the track are displayed with the color gradient from cyan (minimum *D*) to magenta (maximum *D*). (**D**) Histogram of the number of SV40-GM1 bonds involved in the binding of the mobile SV40 on a phospholipid membrane. ©Parveen et al., 2017. Originally published in LANGMUIR. https://pubs.acs.org/doi/pdf/10.1021/acs.langmuir.6b04582 [21].

The initial step of viral infection is the attachment of viruses to the plasma membrane, mediated by a specific interaction between viruses and membrane receptors [4,44]. As the binding affinity/strength of a single virus-receptor bond is often weak, viruses rely on multivalent interactions in which more than one ligand or ligand site of a virus interacts with multiple membrane receptors simultaneously [45,46,47] (Figure 2A). By controlling their multivalent binding, viruses adhere to the plasma membrane, laterally diffuse on the membrane and even switch between attached and detached states. The lateral diffusion of viruses on cell membranes hints that their mobility is linked to the number of virus-receptor bonds. Although multiple studies have discussed the importance of the lateral mobility of viruses for their cellular attachment and uptake [47,48], the number of bonds actually involved in a multivalent binding of viruses has not been correlated with their mobility. Microscopy techniques with a high temporal resolution, combined with analysis methods for quantifying the mobility of single viruses, are prerequisites for this type of study.

The classical method for direct and relatively long-term (>1 s) imaging of the translational motion of single viruses is single particle tracking (SPT). In this method, diffusing particles are imaged in real-time by recording a movie (Figure 2(Bi)). Single particle intensities in each frame are fitted with a 2D Gaussian, and the centroid of the Gaussian is registered as the particle’s position (Figure 2(Bii)). The precision of this position determination can be as little as a few nanometers if the SNR is high enough [49,50,51]. Positions of the particle in consecutive frames are linked to construct a particle track (Figure 2(Biii)). The *D* of the tracked particles is obtained by fitting the corresponding mean square displacement (MSD) versus time-lag plot (Figure 2(Biv)). Here, the MSD is the squared distance travelled by the particle within a time-lag (*∆t*), that is the time between first frame and any later frame. In the simplest case of Brownian diffusion, the MSD shows a linear dependence on time, i.e., *MSD* = 4*D∆t* or 6*D∆t* for 2D or 3D motion, respectively [52,53,54]. Deviation from linearity indicates complex types of motions, such as anomalous/hindered, confined [48,55,56] and super-diffusive/active motion [57,58]. In a dynamic system the particle diffusivity might change over the course of its displacement. For this reason a rolling-window algorithm might be used to allow subtrajectory analysis and to trace transient mobility [59,60,61]. The temporal resolution of the microscope poses an upper limit of *D* values that can be probed with SPT, while the precision of the position determination poses a lower limit on *D* (Table 1). Camera-based imaging techniques have been used for tracking viruses with values for *D* ranging from 10^−5^–1 µm^2^/s [48,57,62].

We used TIRFM to capture the mobility of simian virus 40 (SV40) bound to receptors embedded in a supported phospholipid membrane, and determined the time-dependent *D* of the viruses using rolling-window SPT [21]. The aim was to examine the changes in the multivalent binding of a membrane-bound single virus from its time-dependent lateral mobility. To be able to do this at the temporal resolution of TIRFM, we exploited a receptor-mediated competition which allows an exchange of the receptors from the virus to a competitive ligand. In this way, we managed to extract four different types of SV40 trajectories. The observed heterogeneity is likely relevant to the processes occurring prior to cellular uptake of viruses, such as their recruitment to specific membrane sites or confinement on a membrane [63]. Interestingly, stepwise changes in the *D* of the membrane-bound SV40 within a single trajectory were also detected (Figure 2C). This data directly displays the advantage of single-particle imaging to capture the transient mobility of viruses upon a change in their multivalent binding. According to the analysis, the mean *D* of the mobile SV40 was 0.1 µm^2^/s, ~20 times lower than the corresponding *D* in suspension (~2 µm^2^/s). This indicates that the particles are bound on the lipid-membrane and their lateral mobility is likely controlled by the numbers of SV40-receptor bonds involve in the binding. In line with this, Block et al. have shown that the *D* of nanoparticles tethered to a mobile lipid-membrane is inversely proportional to the number of the tethers [60]. Applying this linear inverse relation to the *D* distribution of SV40 we found that the majority of mobile particles were attached to about 6 receptor molecules, and the SV40 attachment on a lipid membrane may require at least 3 receptor molecules (Figure 2D). In the literature, most SPT studies of viruses focus on the viral transport mechanism from plasma membrane to the virus integration site [48,52,53,57] and are not dedicated to quantify the multivalent interaction viruses on membranes. Our finding that the number of the virus-receptor bonds on a membrane changes dynamically contributes to a better fundamental understanding of this process, and provides a protocol for examining virus mobility on membranes in general.

## 5. Quantitative Confocal Microscopy and Förster Resonance Energy Transfer (FRET) Reveals Dynamic Integrase (IN) Oligomerization

**Figure 3:**
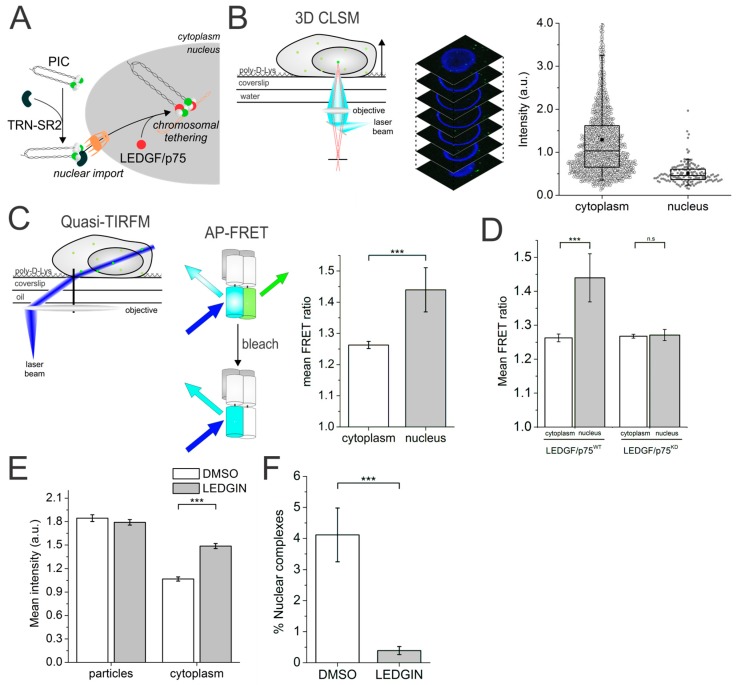
(**A**) Cartoon of the cellular cofactors TRN-SR2 and LEDGF/p75 involved in nuclear import and pre-integration complex (PIC) tethering to the host genome; (**B**) (left) 3D confocal imaging of cells infected with fluorescently labeled viral particles (Right) Fluorescence intensity of HIV_IN-eGFP_ complexes in infected cells in the cytoplasm (black circles) and nucleus (grey circles); (**C**) (left) Quasi-TIRFM imaging of HIV_IN-mTFP1+IN-mVenus_ in infected cells enables acceptor photobleaching FRET measurements. (Right) Mean FRET ratio of HIV_IN-mTFP1+IN-mVenus_ complexes in the cytoplasm and nucleus of infected HeLaP4 cells obtained using acceptor photobleaching FRET and quasi-TIRFM; (**D**) Mean FRET ratio of HIV_IN-mTFP1+IN-mVenus_ in the cytoplasm (white) and nucleus (grey) in wild-type HeLaP4 infected cells and LEDGF/p75 depleted cells showing no increase in nuclear FRET signal in the latter; (**E**) Mean fluorescence intensity of viral particles and viral complexes localized in the cytoplasm of infected cells produced in the presence of DMSO (white) and LEDGINs (grey); (**F**) Percentage of nuclear complexes in cells infected with viral particles produced in the presence of DMSO (white) or LEDGINs (grey). (C-F) *** = *p*-value < 0.001 and n.s. = not significant, as obtained from an unpaired two-sample *t*-test with unequal variance. ©Borrenberghs et al., 2016. Originally published in SCIENTIFIC REPORTS. https://www.nature.com/articles/srep36485 [17].

Nuclear import of the HIV-1 pre-integration complex (PIC), the large nucleoprotein complex composed of double-stranded vDNA, cellular and viral proteins, is a critical yet ill-understood step in viral replication [64,65,66]. Next to the viral components, different cellular cofactors have been reported to be involved in HIV-1 nuclear import and subsequent integration of the vDNA into the host genome [67,68,69,70]. Transportin-SR2 (TRN-SR2), for example, is a karyophilic protein shown to be involved in viral nuclear import [68,71] and LEDGF/p75 is the nuclear cofactor that guides and tethers the PIC to the host genome (Figure 3A) [72,73,74]. How and where exactly these co-factors assist viral replication remains unknown because of the highly asynchronous life cycle of each individual viral particle, rendering a mechanistic study via ensemble methods next to impossible. We opted to use quantitative 3D confocal laser scanning microscopy (CLSM) combined with quasi-TIRFM based FRET imaging to monitor the IN content and quaternary structure, respectively, throughout the pre-integration steps. Quasi-TIRFM (reviewed in Figure S1) allows high-SNR (as opposed to normal widefield) and up to 5 µm deep (as opposed to normal TIRFM) imaging [75].

To evaluate a possible stripping of intasomes at the nuclear border, we first quantified the number of IN-eGFP molecules in a single virus via 3D-CLSM and 2D Gaussian fitting (Figure 3B, left). In this way, we could reveal a 2-fold higher IN content for cytoplasmic complexes compared to their nuclear counterparts (Figure 3B, right). Then, via quasi-TIRFM (Figure 3C, left) we showed a higher FRET signal in the nucleus than in the cytosol (Figure 3C, right). This indicated a change in the oligomeric state of the IN–FP complexes upon nuclear entry of the PICs. To explore the causes of these changes, we depleted or mutated cellular factors such as TRN-SR2, LEDGF/p75, or used LEDGINs (introduced in Section 3) [43,73,76] during viral production. In this way, we could deduce that LEDGF/p75, and not TRN-SR2, orchestrates the IN oligomeric state change (Figure 3D), but not the IN content change. On the other hand, viruses produced in the presence of LEDGINs were unable to lose the necessary number of IN moieties (Figure 3E) at cell entry that would allow efficient nuclear entry (Figure 3F).

To summarize, using our methods we could show the requirement for a PIC IN “uncoating step” during nuclear entry, as well as a role of LEDGF/p75 in changing the oligomerization and/or conformational dynamics of IN in the nuclear PIC. Importantly, analysis of dynamic interactions with FRET would require real-time imaging of viral particles. In recent years, many research groups have been successful in performing real-time imaging of HIV-1 in live infected cells [77,78,79,80,81], and the corresponding colocalization and SPT analyses have provided new insights into the key steps of HIV-1 infection. Along these lines, real-time FRET imaging employing time-resolved confocal microscopy (fluorescence lifetime imaging microscopy and time-resolved anisotropy imaging) might provide a powerful tool to study the dynamics of a specific molecular interaction during the replication cycle of viruses. Finally, the innate ability to genomically integrate its DNA renders retroviruses potential gene therapeutic carriers. One such example is murine leukemia virus (MLV)-based vectors which have been successful in clinical trials [82,83]. However, in a few cases insertional mutagenesis occurred resulting in the development of leukemia [84,85,86]. A better understanding on the integration mechanism of retroviruses in general may help to develop safer vectors for retroviral-based gene therapy. In addition to the HIV-1 integration studies [17], we are establishing a FRET-based assay for single MLV particles to explore the role of specific viral and cellular cofactors in the MLV integration [87].

## 6. Image Correlation Spectroscopy Reveals Cytosolic Assembly of HIV-1 Gag

Upon successful retroviral integration, the encoded viral components are expressed by the host’s cellular machinery. These components assemble in the cytosol and/or at the plasma membrane to bud off as progenies. In the case of HIV-1, a key player of this assembly process is the Gag polyprotein comprised of the major structural viral proteins capsid (CA), matrix (MA), nucleocapsid (NC) [88,89]. Mutations in the structural protein units of HIV-1 Gag can impair the synthesis of fully functional viruses [89,90,91,92,93]. HIV-1 Gag proteins are expressed in the cytoplasm and transported to the plasma membrane where they are known to multimerize to initiate viral assembly [79,94]. This makes HIV-1 Gag, in particular its multimerization process, a potential target of antivirals [95,96]. HIV-1 Gag literature is dominated by Gag assembly at the plasma membrane [97,98,99], but cytosolic Gag is not well characterized. A few in vitro studies [100,101] and characterization of other retroviral Gag [15,102] have indicated Gag assembly nucleation in the cytoplasm. However, these studies lacked any detailed insights. Molecular interactions are key to any nucleation process. A classic method for examining molecular interactions using dual- or multicolor microscopy are the so-called colocalization methods. Colocalization, whether via super-resolution microscopy or not, is most often performed on fixed samples. When the suspected interacting molecules are mobile, as often is the case for live samples, these methods might provide false-positive or -negative results. Also, the translational mobility of a molecule or particle itself informs on possible underlying molecular interactions. Methods designed to analyze diffusion and even co-diffusion of the two molecules are dual-color SPT [80,103,104,105] and fluctuation-based correlation spectroscopy, the latter being less demanding than SPT in terms of required dye brightness and photostability.

**Figure 4:**
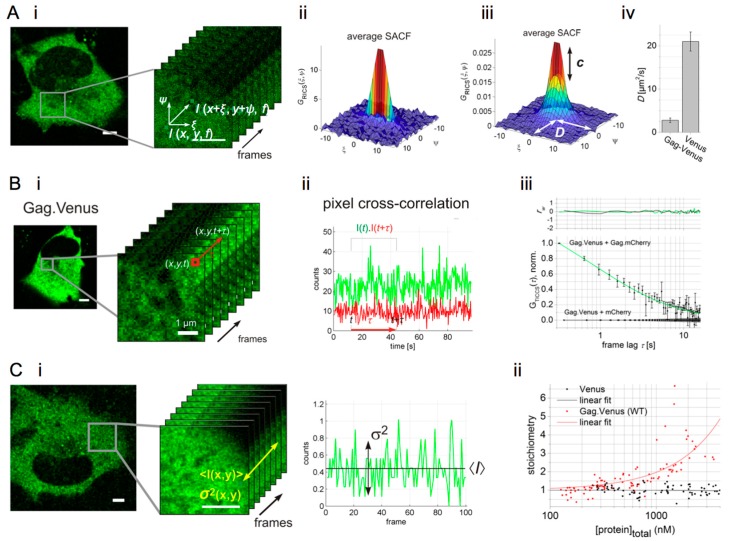
(**A**) (**i**) A confocal image of cytosolic Gag tagged with the Venus fluorescent protein (FP). Spatial correlation of an area of the image is performed via raster image correlation spectroscopy (RICS) analysis, where multiple scanned frames are used to obtain an average spatial correlation; (**ii**) average spatial autocorrelation function (SACF) of fast diffusing molecules, here cytosolic Venus FP; (**iii**) average SACF of slowly diffusing molecules and a fit to the SACF model provide concentration (*c*) and diffusion coefficient (*D*) of the molecules. Here, Gag.Venus data is shown; (**iv**) *D* of cytosolic Gag.Venus versus free cytosolic Venus determined from the RICS analysis. (**B**) (**i**) Confocal image series and temporal correlation via TICS of a pixel signal in the series; (**ii**) Exemplary temporal cross-correlation of dual-color signals determined via TICCS analysis; (**iii**) TICCS analysis of the co-expressed Gag.Venus and Gag.mCherry and the corresponding negative control (Gag.Venus+mCherry). *r*_w_ is the weighted residual of the fit. (**C**) (**i**) Intensity map of cytosolic Gag.FP, illustrating the mean intensity and variance of the signal in a single pixel; (**ii**) mean stoichiometry of diffusing cytosolic molecules as a function of their concentration. ©Hendrix et al., 2015. Originally published in JOURNAL OF CELL BIOLOGY. http://jcb.rupress.org/content/210/4/629 [19].

Correlation spectroscopy groups a number of methods that extract dye brightness, concentration and mobility information from fluctuating fluorescence signals that are recorded at roughly 1–1000 nanomolar concentrations on a confocal microscope. Mathematically, a temporal or spatial autocorrelation function (ACF) is calculated from the fluorescence signal, which displays the self-similarity of the fluorescence signal [106]. Molecular parameters are then determined by fitting the ACF with a suitable model function that renders the diffusivity *D* and the absolute concentration [107,108,109]. The classic temporal correlation method is fluorescence correlation spectroscopy (FCS), suitable for probing fast molecular motion (1–1000 µm^2^/s). Similarly, a spatial correlation can be calculated using images acquired with CLSM via so-called raster image correlation spectroscopy (RICS). In fact, CLSM images can be seen as a time-dependent fluorescence measurement, where each time point is measured at a different location in space (Figure 4(Ai)). When recorded in solution or in subcellular compartments of live cells at appropriate settings (~50 nm pixel size, oversampling the lateral resolution by at least twofold, ~20 µs pixel dwell time, ~5 ms line time and ~1 µW laser power in solution) diffusing molecules cause a peculiar correlation between adjacent pixels in the image. If molecules diffuse fast (~50–500 µm^2^/s), correlation is mostly, or only seen in the faster *x*-scanning direction (exemplified in Figure 4(Aii)). If molecules diffuse slowly (~0.1–10 µm^2^/s), correlation is still seen in the *x*-scanning direction due to the spatial oversampling, but, more interestingly, also in the slower *y*-scanning direction (exemplified in Figure 4(Aiii)). Correlation methods can also be extended to two or more imaging channels, to study the co-diffusion of differently labeled molecules. In this way fluorescence cross-correlation spectroscopy (FCCS) [110] and cross-correlation (cc)RICS [12,111], for example, can be used to quantify the interaction affinity of two molecules, each labeled with a different fluorophore (Table 1). In cross-correlation spectroscopy, the application of pulsed interleaved excitation (PIE) is quite useful for delivering fluorescence crosstalk-free analyses (reviewed in Figure S3). Correlation methods have been applied before in virus research, for example to detect HIV-1 RNA [112], to quantify the Gag stoichiometry in viral particles [113], to study assembly of HTLV-1 Gag in the cytoplasm [114] and to study interactions between viruses and cellular cofactors [115]. In our research, we imaged Gag by conjugating it to the FPs Venus (yellow) and mCherry (red). These FPs were chosen because of their fast folding and high maturation rates. For cytosolic HIV-1 Gag.Venus, we found using single-color RICS that ~60% of the protein was mobile (*D* = ~2.4 µm^2^/s), yet significantly slower than free cytosolic Venus (Figure 4(Aiv)), or than expected for freely diffusing cytosolic Gag [19]. This reduced mobility did not correspond to an oligomeric state of Gag.FP, as confirmed from a corresponding spatial two-color cross-correlation (PIE-ccRICS) analysis.

The mobility of some protein complexes in cells and cellular compartments can be quite low (*D* of 10^−1^–10^−3^ µm^2^/s), rendering these species seemingly immobile to methods such as FCS and RICS. In such cases, correlating pixels through time (rather than through space) over the course of an image series allows correlation analyses on much longer timescales, with a method referred to as temporal image correlation spectroscopy (TICS) or ‘imaging FCS’ [116,117,118] (Figure 4(Bi)). To dig deeper into the Gag imaging data, we investigated the seemingly “immobile” Gag fraction using TICS. Indeed, single-color TICS analysis revealed a second, much slower Gag fraction (D = ~0.014 µm^2^/s) which was still well above the expected microscope drift or cellular/organelle motion (10^−4^ µm^2^/s) and attributed to a confined motion of a fraction of Gag.FP molecules. Dual-color PIE-TICCS analysis (Figure 4(Bii)) subsequently confirmed this slow-moving Gag.FP fraction corresponded to an oligomerized Gag.FP fraction (Figure 4(Biii)) [19].

Finally, the molecular stoichiometry of an oligomeric complex or quaternary structure can be determined from the ratio of the molecular fluorescence brightness of the complex to the brightness of the monomer. The number and brightness (N&B) analysis method is an “easy” alternative to correlation methods to quantify molecular brightness, and thus stoichiometry. It works by simply measuring the average and variance of confocal fluorescence signals [11,119] (Figure 4(Ci)). Applied to Gag, we could see the stoichiometry of the Gag.FP oligomers escalate from 1 to 4 with increasing Gag concentration (up to 2.5 μM), suggesting a micromolar affinity and open polymerization process of cytosolic Gag.FP monomers (Figure 4(Cii)). Combining the results from RICS, TICS, cross-correlation and N&B analysis of wild-type and mutated HIV Gag, we could establish a model describing the cytosolic nucleation of the Gag assembly process for the first time.

## 7. Super-Resolution Microscopy Sheds Light on Viral Restriction at the Plasma Membrane

**Figure 5:**
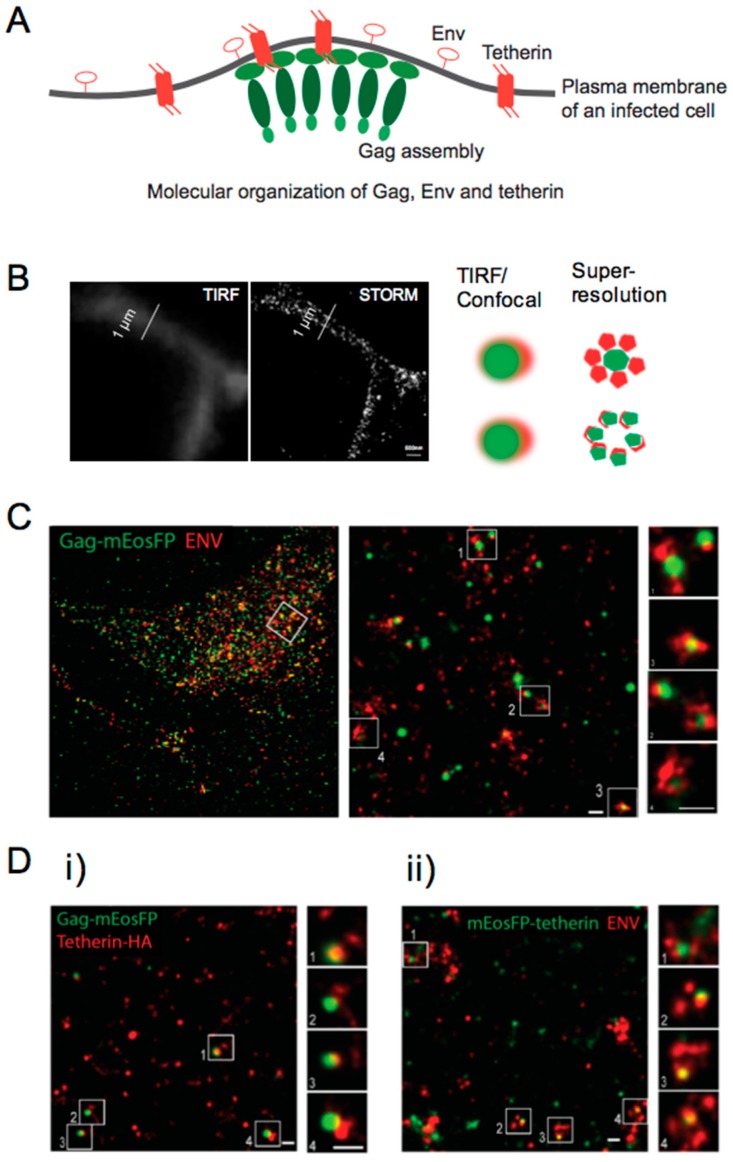
(**A**) Scheme of molecular organization of HIV-1 proteins, Gag and Env and a molecular restriction factor for Gag assembly, i.e., tetherin. A super-resolution microscopy technique was applied which is able to resolve the corresponding structure/organization; (**B**) images of densely distributed fluorescent molecules acquired with TIRFM (diffraction-limited) and super-resolution microscopy techniques. Schemes of “true” and “false” colocalization of molecules detected with diffraction-limited and super-resolution microscopy techniques; (**C**) HeLa cells expressing HIV ∆Vpu and Gag-mEos (green) were labeled using primary/secondary antibody against gp120 (Alexa Fluor 647, red). The middle and right panels are magnifications of the areas indicated by the white squares in the left and middle panels, respectively; (**D**) representative regions of super-resolution images of HeLa cells transfected with HIV-1 ∆Vpu and (**i**) Gag-mEosFP and tetherin-HA; (**ii**) Gag-mEosFP and tetherin-Flag. Tetherin-HA and HIV-1 Env were stained by indirect immunofluorescence for HA and Env, respectively. The right panels are magnifications of the areas indicated by the white squares in the left panels. Scale bar of the images is 200 nm. ©Lehmann et al., 2011. Originally published in PLOS PATHOGENS. https://doi.org/10.1371/journal.ppat.1002456 [20].

After hijacking the protein expression machinery of the host cells, new virus particles are formed and discharged into the extracellular environment, promoting the propagation of the virus. In order to enter new cells, the HIV encodes the surface-expressed viral protein Env, a 160-kD glycoprotein (gp160). After translation, gp160 is cleaved by cellular proteases into the surface proteins gp41 and gp120. The latter is responsible for the interaction between HIV particles and the CD4 receptors present at the membrane of host cells. The assembly of new particles at the plasma membrane is one of the final steps of the viral replication cycle. The two HIV envelope glycoproteins, gp120 and gp41, are transported to the plasma membrane of the host cell while HIV Gag associates with the inner surface of the plasma membrane. The molecular mechanisms behind HIV-1 assembly have been explored by following the recruitment and accumulation of Gag at the plasma membrane [97]. Methods like mobility analysis or FRET provide information concerning the dynamic molecular interaction during/at assembly, but cannot probe molecular organization which, in a way, allows a direct visual inspection of possible interactions (Figure 5A).

Objects that are closer together than 200 nm will always exhibit colocalization in a conventional diffraction-limited microscope, irrespective of a direct interaction. Super-resolution fluorescence microscopy can overcome this shortcoming by imaging molecules with a much-improved resolution (down to ~20–30 nm) (Figure 5B). From the panoply of super-resolution methods available, the most used methods for imaging biological samples are the single molecule-based methods, namely photo-activation localization microscopy (PALM) and stochastic optical reconstruction microscopy (STORM). These methods use standard wide-field microscopes with single molecule sensitivity and exploit the switching properties of some fluorophores to discriminate single emitters in time. In order to retrieve a super-resolved image, we acquire time-lapse fluorescence images where each frame captures a different subset of molecules in the “on” state. Fitting the fluorescence signal of each molecule with a 2D Gaussian allows to localize the individual fluorophores with a high precision and accuracy. A super-resolved fluorescent image is then reconstructed from the calculated positions of all detected fluorophores. A typical PALM/STORM image is rendered from 5,000–20,000 frames (around 3–15 min). Despite the low temporal resolution achievable with these methods, the high spatial resolution has, for example, provided new insights into the structure of virus assembly sites at the plasma membrane [120]. For a complete overview of the super-resolution fluorescence microscopy methods available, see reference [121].

We investigated the assembly of HIV Gag at the plasma membrane and the effects of tetherin using super-resolution fluorescence microscopy [20]. In order to perform single molecule-based super-resolution microscopy, the protein of interest needs to be labeled with a photo-switchable fluorophore. For biological samples, photo-switchable FPs are often used. Due to its brightness and photo-switchable properties, mEosFP and the improved mEos3.2 version are amongst the most suitable FPs used in PALM. mEosFP originally emits fluorescence in the green region of the spectra and, upon ultraviolet (UV) illumination, a β-elimination reaction in the chromophore shifts the absorption and emission bands into the red region [122,123]. Since mEosFP emits in both the green and red spectral range, multi-color super-resolution is best performed in combination with a far-red emitting fluorophore. At the moment of this writing, far-red Alexa Fluor 647 displays the best photo-switching properties for single molecule-based super-resolution microscopy [124].

Super-resolution images of HIV assembly revealed important structural features of viral budding, which previously could only be obtained by electron microscopy. As depicted in Figure 5C, HIV Gag-mEosFP clusters of varying sizes were found co-localizing with clustered Env at the plasma membrane. Super-resolution imaging was extended to tetherin, a cellular restriction factor that inhibits the release of several enveloped viruses [125,126]. To counteract the action of tetherin, viruses have developed several anti-tetherin activities. In the specific case of HIV, the restriction of virus release by tetherin is neutralized by the Vpu viral protein, which degrades and removes tetherin from the surface of the host cell. We found that tetherin is homogenously distributed in clusters of 70–90 nm at the plasma membrane of HeLa cells. The interaction of tetherin with viral proteins was evaluated using Vpu deficient HIV (HIV ∆Vpu). Budding structures were mostly found in close proximity with a single cluster of tetherin (Figure 5D), which suggested that the clustering of tetherin molecules is necessary for efficient viral restriction. Since photoswitching of mEosFP results from an irreversible reaction, each detectable emissive state can be accurately assigned to a single molecule. A detailed characterization of the photophysical properties of mEosFP allowed the quantification of the number of tetherin molecules in each cluster, revealing 4–7 and 5–11 tetherin dimers, in the presence and absence of HIV, respectively. Our data suggests that pre-existing tetherin clusters can associate with assembling HIV particles, where approximately 70% of the molecules may participate in restricting HIV release.

This was the first time that super-resolution fluorescence microscopy was applied to study the structure and interaction of viral proteins at the cell membrane. In the absence of Vpu, the release of the newly formed particles is restricted by the action of tetherin, pre-clustered at the plasma membrane. The reorganization of viral proteins, membranes and membrane components is involved in most steps of virus replication, and quantifying these processes can provide unique insights into the underlying molecular mechanisms. Although x-ray crystallography and electron microscopy are still the most powerful techniques to resolve molecular structure, dual- or multi-color super-resolution microscopy imaging has the advantage of being performed at near-native conditions. Over the years, virologists have, therefore, become increasingly interested in resolving/imaging the molecular structure/organization of viruses or viral-complexes via super-resolution fluorescence microscopy, in particular the viral attachment and assembly process [127,128,129]. For an extended overview of the applications of super-resolution fluorescence microscopy in virus studies, see references [8,120,130].

## 8. General Conclusions and Outlook

Single-molecule imaging techniques are powerful tools to shed light on the different steps in viral replication cycles with high temporal and/or spatial resolution. While reviewing the case studies, we addressed the benefits of the different fluorescence modalities for quantitative analysis of the molecular processes used. Still, many aspects of these imaging approaches can be improved for a more accurate and semi-automated determination of the molecular parameters in their native conditions. There is a constant development of the labeling methods of viruses [131], for example, using tetra-cysteine tags [132], non-natural amino acids [133] and FPs [134,135] suitable for live-cell and super-resolution imaging. Then, the algorithms used for the analysis of SPT, smFRET, correlation methods and super-resolution imaging could be more unified, user-friendly and easily accessible. In this respect, many interesting open-source tools for advanced image analysis have recently appeared [136,137,138,139,140]. Finally, video-rate super-resolution techniques like 3D-STORM [141] and single-molecule tracking PALM (sptPALM) [142,143] applicable for live-cell imaging are promising for resolving the molecular mobility and structure of the viral complex or assembly simultaneously. We think the future of single virus imaging lies in exploiting the multidimensionality of the fluorescence signal as a handle for gaining the maximum amount of information from a single measurement, and in combining this with real-time imaging of single viral complexes. In doing so, hopefully novel insights into the molecular mechanisms governing viral replication will be obtained that will aid the development of new HIV targeting drugs and virus-based gene therapy.

## Figures and Tables

**Table 1:** List of quantitative fluorescence methods and the determined parameters. A full description of the listed modalities is provided in the Supporting information.

Method	Modality	Quantities	Range
Fluorescence correlation spectroscopy (FCS)	Confocal microscope, no scanning	Diffusion coefficient	1–1000 µm^2^/s
		Molecular concentration	1–1000 nM
		Rel. molecular mass (aqueous buffer)	0.5–1000 kDa
		Stoichiometry	any, if monodisperse
		Dissociation constant	1–1000 nM
Raster image correlation spectroscopy (RICS)	CLSM, PIE-CLSM, scanning disk (SD)-CLSM	Diffusion coefficient	1–1000 µm^2^/s
		Molecular concentration	1–1000 nM
		Rel. molecular mass (aqueous buffer)	0.5–1000 kDa
		Stoichiometry	any, if monodisperse
		Dissociation constant	1–1000 nM
Temporal image correlation spectroscopy (TICS)	CLSM, PIE-CLSM, TIRFM	Diffusion coefficient	0.001–10 µm^2^/s
		Molecular concentration	1–1000 nM
		Stoichiometry	any, if monodisperse
		Dissociation constant	1–1000 nM
Single particle tracking (SPT)	TIRFM, SD-CLSM	Diffusion coefficient	10^−5^–10 µm^2^/s
Dual-color SPT			
Cross-correlation (fluorescence cross correlation spectroscopy (FCCS), TICCS, ccRICS)	Confocal, CLSM PIE-Confocal, PIE-CLSM	Stoichiometry	any, if monodisperse
		Diffusion coefficient	1–1000 µm^2^/s (FCCS, ccRICS), 0.001–10 µm^2^/s (TICCS)
		Binding constant	nM to µM
Förster resonance energy transfer (FRET)	Wide-field	Molecular distance	1–10 nm
Single-molecule FRET (smFRET)	TIRFM, Confocal, CLSM, PIE-Confocal, PIE-CLSM	Structure	1 Å precision
Photo-activation localization microscopy (PALM)/stochastic optical reconstruction microscopy (STORM)	Wide-field, TIRFM	Structure information	20–30 nm precision 20–30 nm precision
		Colocalization	
Number and brightness (N&B)	Confocal, CLSM PIE-Confocal, PIE-CLSM	Molecular concentration	1–1000 nM
		Stoichiometry	any, if monodisperse

## Supplementary Materials

The following are available online at http://www.mdpi.com/1999-4915/10/5/250/s1, Figure S1: Modalities of wide-field microscopy, Figure S2: Continuous-wave confocal imaging microscopy, Figure S3: Pulsed interleaved excitation confocal imaging microscopy.
