# Supplementary material for: Single Viruses on the Fluorescence Microscope: Imaging Molecular Mobility, Interactions and Structure Sheds New Light on Viral Replication
Source: Viruses. 2018 May 10;10(5):250. doi: 10.3390/v10050250 (PMC5977243; doi:10.3390/v10050250)
Supplement: Supplementary file 1 [file viruses-10-00250-s001.zip › viruses-287770-SI.pdf]

Supporting Information

Single viruses on the fluorescence microscope: imaging molecular mobility, interactions and structure sheds new light on viral replication

Nagma Parveen [1], Doortje Borrenberghs [1], Susana Rocha [1] and Jelle Hendrix [1,2,*]

[1] Laboratory for Photochemistry and Spectroscopy, Molecular Imaging and Photonics Division, Chemistry Department, KU Leuven, B-3001 Leuven, Belgium;
[2] Dynamic Bioimaging Lab, Advanced Optical Microscopy Centre and Biomedical Research Institute (BIOMED), Hasselt University, B-3590 Diepenbeek, Belgium;
* Correspondence: jelle.hendrix@uhasselt.be; Tel.: +32 (0) 11 269213

Received: date; Accepted: date; Published: date

<u>Table of contents</u>

1. Wide-field imaging **2**

Wide-field microscopy 2

Total internal reflection fluorescence microscopy (TIRFM) 2

Quasi-TIRFM 3

2. Confocal imaging **3**

Confocal laser scanning microscopy (CLSM) 3

Spinning disk CLSM 4

3. Confocal imaging using pulsed interleaved excitation **5**

Supporting references **7**

1. Wide-field imaging

Wide-field fluorescence imaging is the illumination and fluorescence imaging of a large (~50×50 μm^2) region-of-interest in the sample. In a typical single-molecule sensitive wide-field fluorescence microscope (Figure S1A), the excitation beam's profile can be cleaned up using a single-mode fiber or pinhole, the beam is expanded to 1-2-inch diameter, possibly homogenized using an opaque element, and then focused at the objective's back focal plane using a high-quality achromatic lens (Figure S1B). If different lasers (each typically ~50-200 mW) are used simultaneously, they can be alternated on the microsecond timescale using the AOTF or mechanical shutters placed in the excitation pathway. Sequential illumination is crucial for dealing with emission crosstalk in multi-color imaging (for a detailed explanation on crosstalk, the reader is referred to section S3).The light exits the objective collimated, with the diameter of the beam entering the objective determining the diameter of the illuminated region in the sample. A power density in the sample of ~1-2000 W/cm^2 per laser is used. Popular cameras for single-molecule imaging are scientific complementary metal oxide semiconductor (sCMOS) cameras or electron-multiplying charge-coupled devices (EMCCD), with the latter providing a good signal, even for dimly fluorescent samples. An extra beam expander (*e.g.* 3.3× for a 60× objective lens) is typically placed in between the microscope's tube lens and the camera to achieve an optimal magnification. Indeed, according to the Nyquist theorem, a 60×3.3 total magnification oversamples the diffraction-limited spatial resolution (d = 200-300 nm) by more than twofold on the camera (16 μm physical pixel size).

One of the main advantages of camera-based microscopy is the relatively high frame rate (roughly ~30 and 100 Hz at full-EMCCD-chip and 4-megapixel sCMOS imaging, respectively). Such video-rate imaging means that dynamic processes occurring on a timescale of 10-30 ms or slower are directly visible and can be quantified; anything faster than this will be averaged out ('blurred') over (regions in) the recorded image series, although this effect can be countered by stroboscopic illumination [6]. Because of its optimal frame rate, camera-based microscopy has been quite popular in the single virus imaging field, for example, to study translational mobility of viruses on plasma membrane and in cells [7-9], to probe the kinetics of virus attachment-detachment [10,11], and quantify viral assembly at the plasma membrane [12-15].

The quality with which single molecules are 'seen' using fluorescence microscopy is expressed as the signal-to-noise ratio, or simply SNR. In 'normal' wide-field microscopy, out-of-focus fluorescence from regions above and below the objective lens focal plane decreases the experimental SNR below a critical value of ~5, which means >20% of the total registered signal is, in fact, noise. A popular way to increase the SNR is to image the sample via total internal reflection fluorescence microscopy (TIRFM) (Figure S1C). The optical scheme for TIRFM is similar to the one for wide-field microscopy, with the difference that the excitation light is focused off-center from the optical axis at the objective lens' back focal plane. This way, the excitation light exits the objective at an angle, rather than along the optical axis. At the glass-solution interface, the light is completely reflected back instead of being refracted into solution, when the incident angle is ≥ the critical angle ([illegible]$_c$) given by Snell's law of refraction, *i.e.*, ~61° for glass-water (see Figure S1C). At this interface, an evanescent field is generated, *i.e.*, part of the excitation light penetrates the sample and can excite fluorophores close to the glass-solution interface. The penetration depth of this evanescent field is only ~100-200 nm, decays exponentially along the optical axis, and depends both on the wavelength of the light, and the angle of incidence [16]. In other words, the fluorescence is restricted to a very thin region close to the coverslip surface, thereby drastically increasing the SNR up to 10 or more. This renders TIRFM a method of choice for single-molecule imaging. Many single virus imaging studies have already been performed using TIRFM and they provide more insight into virus-membrane attachment [17], viral assembly [18], viral fusion [19] or viral cell entry [20]. We typically design our microscopes to allow easy switching between wide-field and TIRF modes, although simultaneous wide-field and TIRF imaging is also possible using synchronized electronic shutters [21]. The latter has been used in HIV research to visualize viral assembly at the plasma membrane while simultaneously monitoring cytosolic components. In the main text, we have discussed our

investigations using TIRFM on immobilized viruses (Figure 1A in section 3 of the main text) [2], virus-membrane attachment (section 4) [5] and viral budding (section 7) [4].

As TIRFM is limited to imaging processes close to the microscope coverslip, this inhibits its application for single molecule or single viral imaging inside 3D specimens such as cells and cellular compartments. To balance the high SNR offered by TIRFM and the possibility of imaging molecules in three dimensions offered by normal wide-field microscopy, highly inclined and laminated optical sheet (HILO) [22] was developed, also referred to simply as 'quasi-TIRFM'. In quasi-TIRFM, the excitation light incidents at the glass-water interface at an angle slightly below the critical angle (θ_c). This causes the excitation light to refract into the sample in the shape of a thin 1-2 μm 'sheet' (Figure S1D). Since molecules outside the sheet are hardly excited, the SNR is considerably better than for normal wide-field (around 5-10). In section 5 of the main text, we have reviewed our application of quasi-TIRF to study protein interactions within subcellular viral complexes [1].

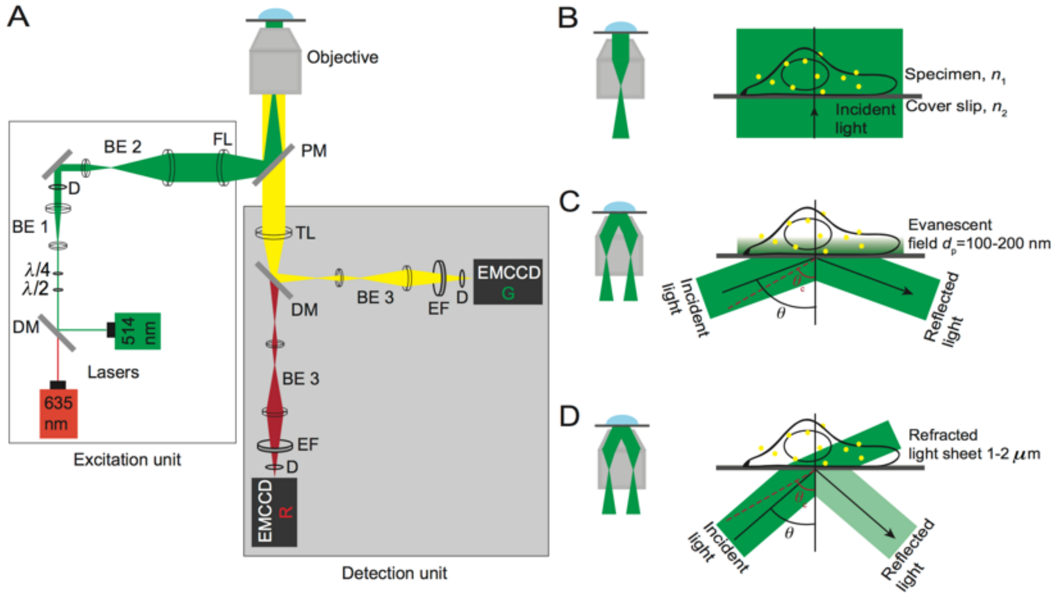

Figure S1. A) Scheme of a dual-color wide-field microscope with illumination profiles allowing B) wide-field, C) TIRF and D) quasi-TIRF microscopy. DM = dichroic mirror, $\lambda/2$ = half-wave plate, $\lambda/4$ = quarter-wave plate BE = beam expander consists of two planoconvex lenses, D = diaphragm (regulates the amount of light passing through), FL = focusing lens, PM = polychroic mirror, TL = tubes lens, EF = emission filter, EMCCD = electron-multiplying charge-coupled device, d_p = penetration depth of evanescent field, n = refractive indexes and $n_1 > n_2$.

2. Confocal imaging

In confocal laser scanning microscopy (CLSM), the registered fluorescence originates from only a very small (~1 μm^3) excited region in the sample, and images are made by scanning the laser beam through the sample in a raster-scanning mode and recording the photons pixel by pixel. In a typical confocal microscope (Figure S2), Gaussian distributed and perfectly concentric excitation beams are obtained by combining all lasers into a single-mode optical fiber. The output of the fiber is collimated by an apochromatic collimating lens and sent through a pair of closely spaced galvanometric *x-y* scanning mirrors (also termed 'galvo'). A beam expander projects the mid-point between the mirrors onto the back focal plane of the objective lens, and ensures the latter is slightly overfilled with light. As opposed to wide-field fluorescence, the objective lens now focuses all the incident light into a single diffraction-limited focal point, with dimensions given by the Rayleigh criterion. As the fluorescence goes back through the galvo, the resulting beam can be focused by the microscope's tube lens through a 50-100-μm diameter immobile pinhole, that efficiently rejects out-of-focus light. Finally, the light is detected on a point detector such as a photomultiplier tube (PMT) or a more sensitive avalanche photodiode (APD). Images are finally built up by scanning the excitation spot

pixel- and line-wise over the sample. The excitation power density in confocal microscopy needed for good a SNR is typically somewhat higher (0.3-3 kW/cm^2) than for wide-field microscopy because (i) the pinhole rejects some in-focus light, (ii) generally, more optics are between the sample and detector, (iii) detectors for wide-field microscopy are still superior in terms of detection efficiency, and (iv) it ensures enough photons can be acquired in the little time the excitation spot spends in each pixel (typically around 20 μs, which is some 1000-fold smaller than for wide-field microscopy). Because of the focused excitation and the optical sectioning by the pinhole, confocal microscopy generates sharp optical slice of the imaged sample. Moreover, if image stacks are recorded with each slice partially overlapping along the optical axis (the *z* dimension), confocal microscopy can be used to generate a 3D reconstructed image of the specimen. Confocal microscopy has been popular in virus research for localizing single viruses in different compartments of fixed and live cells either at different time of infection or in real-time. The majority of these studies are focused on the viral spatial distribution and colocalization with other viral/cellular components, providing insights into their role in processes such as nuclear import of viruses [23-25]. We have used confocal microscopy to localize labeled human immunodeficiency virus type 1 (HIV-1) inside cells [2] and to assess the number of fluorescently labeled integrase enzyme units inside subcellular viral complexes (section 5 of the main text) [1].

The drawback of the laser scanning process in CLSM is that the achieved framerate is only on the order of 1 Hz, hence only very slow dynamic processes can be directly visualized. Using image correlation spectroscopy, however, biophysical properties of faster dynamic processes that are blurred in the obtained images can still be quantified accurately (see section 6 of the main text). On the other hand, the imaging framerate can be improved considerably by using a spinning disk (SD) CLSM [26]. Here, the specimen is simultaneously illuminated at multiple points using two laterally aligned disks, one holding an array of micro-lenses, the other the associated pinholes. By spinning the disk, an array of collimated focused laser beams scans the specimen. The emitted photons from the scanned points are simultaneously detected using one or more EMCCD cameras. SD-CLSM has been used successfully for the real-time tracking of viruses in cells [7,27].

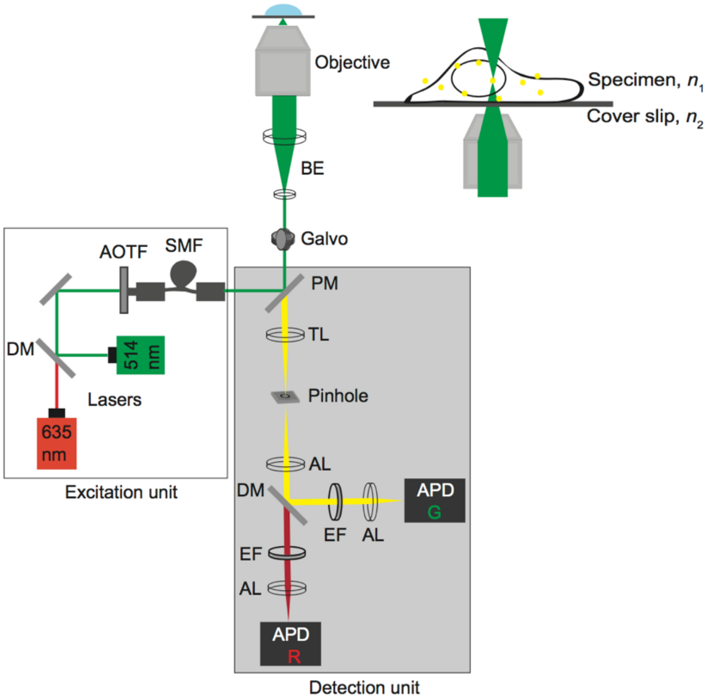

Figure S2. Scheme of a dual-color continuous wave (CW) CLSM with illumination profile of a confocal microscopy. Here, DM = dichroic mirror, AOTF = acousto-optical tunable filter SMF = single-mode optical fiber, BE = beam expander consists of two planoconvex lenses, PM = polychroic mirror, TL = tubes lens, AL= achromatic lens (reduces chromatic aberration), EF = emission filter, APD = avalanche photodiode.

3. Confocal imaging using pulsed interleaved excitation

Multicolor fluorescence microscopy suffers from spectral emission crosstalk or bleed-through, the leakage of a blue-shifted (lower wavelength) fluorophore's emission into the red-shifted fluorophore's detection channel. This problem is classically dealt with by sequentially exciting the sample with each used laser every other image frame (or even every other image line in modern confocal microscopes), and is commonly referred to as alternating laser excitation (ALEX) [28]. One step further is pulsed interleaved excitation (PIE), or alternating excitation on the nanosecond timescale. The advantage of PIE over standard ALEX is the ability to probe also very dynamic processes (μs-ms timescale) accurately with multicolor microscopy [29,30]. Although the optical scheme of a typical PIE-CLSM appears similar to that of a normal CLSM at first sight, there are some crucial differences (Figure S3A). PIE employs so-called time-correlated single photon counting (TCSPC) [31], a method that allows timing photons relative to the moment the fluorescent molecule was excited by the laser. Doing so requires (i) ultrafast pulsing (in picoseconds) lasers instead of normal, continuous wave lasers, (ii) detectors that can operate in the 'single-photon counting' mode and (iii) picosecond resolution photon timing electronics that synchronize all lasers and detectors. A PIE-CLSM is commercially available as a standalone instrument, but a normal CLSM can nowadays also be upgraded without much ado, and literature exists on how to build a PIE-CLSM for the more adventurous virologist [30,32,33].

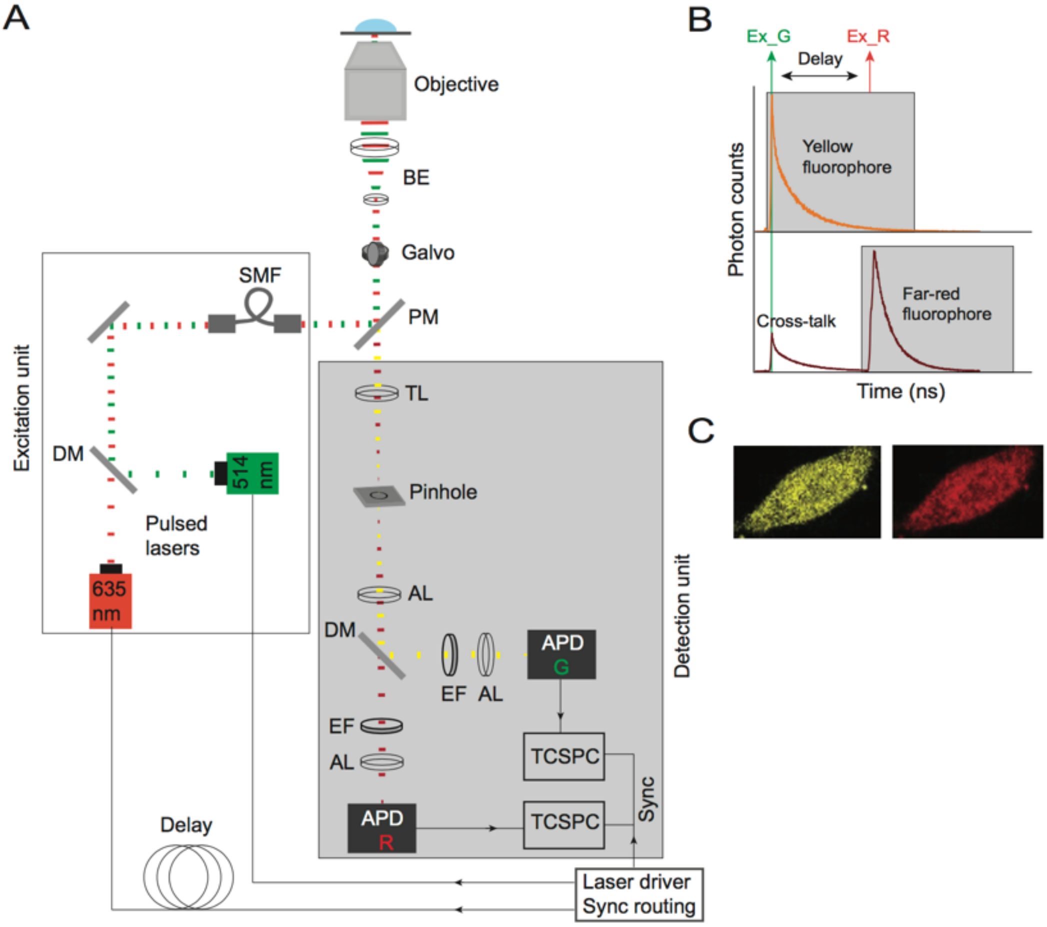

Figure S3. A) Scheme of a PIE-CLSM with pulsed laser sources and a TCSPC device in addition to the optical setup of the CLSM illustrated in Figure S2. The red excitation pulses interleave the green ones and they are presented with the broken lines. B) Illustration of a two-color excitation and TCSPC detection (fluorescence decay) experiment in a PIE-CLSM microscope. C) Cross-talk free species-selective images obtained from the fluorescence decay of the selected time gates (*gray* areas in Figure S3B). Here, DM = dichroic mirror, SMF = single-mode optical fiber, BE = beam expander consists of two planoconvex lenses, PM = polychroic mirror, TL = tubes lens, AL= achromatic lens (reduces chromatic aberration), EF = emission filter, APD = avalanche photodiode.

The raw data obtained from a two-color PIE-CLSM displays the fluorescence decay of each imaged fluorophore (Figure S3B). In the red-shifted fluorophore's detection channel, the blue-shifted fluorophore's crosstalk is shifted in time relative to the direct emission of the red fluorophore (see Figure S3B). This picosecond timestamp of the data is then used to discard the crosstalk *a priori*, before rendering clean two-color images and image series devoid of crosstalk (Figure S3C). We used PIE-CLSM to study cytosolic assembly of the HIV structural protein Gag before visible membrane attached *punctae* (section 6 of the main text) [3].

The fluorescence decay, as obtained on a PIE-CLSM microscope, reports on the fluorophore's direct environment, and is mostly independent of the fluorophore's concentration. It can be analyzed to sense structural changes of the molecule to which the fluorophore is attached, to probe molecular interactions via Förster resonance energy transfer (FRET) [34], or to simply provide contrast in microscopy images that are otherwise information-poor, to name just a few applications [35,36]. For a more detailed description of fluorescence lifetime imaging microscopy (FLIM), the reader is referred to the literature [37,38]. When combined with polarization optics, the fluorescence emission anisotropy can be determined in addition to the fluorescence intensity and lifetime. The anisotropy reports on rotational diffusion of the excited fluorophore within its fluorescence lifetime, but can also be used to quantify molecular interactions [39]. Single-color or PIE-CLSM imaging of the fluorescence intensity, lifetime and anisotropy is also termed multi-parameter fluorescence imaging spectroscopy (MFIS) [40].

Supporting references

1. Borrenberghs, D.; Dirix, L.; De Wit, F.; Rocha, S.; Blokken, J.; De Houwer, S.; Gijsbers, R.; Christ, F.; Hofkens, J.; Hendrix, J., *et al.* Dynamic oligomerization of integrase orchestrates hiv nuclear entry. *Sci Rep* **2016**, *6*, 14, 10.1038/srep36485.
2. Borrenberghs, D.; Thys, W.; Rocha, S.; Demeulemeester, J.; Weydert, C.; Dedecker, P.; Hofkens, J.; Debyser, Z.; Hendrix, J. Hiv virions as nanoscopic test tubes for probing oligomerization of the integrase enzyme. *ACS Nano* **2014**, *8*, 3531-3545, 10.1021/nn406615v.
3. Hendrix, J.; Baumgartel, V.; Schrimpf, W.; Ivanchenko, S.; Digman, M.A.; Gratton, E.; Krausslich, H.G.; Muller, B.; Lamb, D.C. Live-cell observation of cytosolic hiv-1 assembly onset reveals rna-interacting gag oligomers. *J. Cell Biol.* **2015**, *210*, 629-646, 10.1083/jcb.201504006.
4. Lehmann, M.; Rocha, S.; Mangeat, B.; Blanchet, F.; Uji-i, H.; Hofkens, J.; Piguet, V. Quantitative multicolor super-resolution microscopy reveals tetherin hiv-1 interaction. *PLoS Pathog.* **2011**, *7*, e1002456, 10.1371/journal.ppat.1002456.
5. Parveen, N.; Block, S.; Zhdanov, V.P.; Rydel, G.E.; Hook, F. Detachment of membrane bound virions by competitive ligand binding induced receptor depletion. *Langmuir* **2017**, *33*, 4049-4056, 10.1021/acs.langmuir.6b04582.
6. Flors, C.; Hotta, J.-i.; Uji-i, H.; Dedecker, P.; Ando, R.; Mizuno, H.; Miyawaki, A.; Hofkens, J. A stroboscopic approach for fast photoactivation−localization microscopy with dronpa mutants. *Journal of the American Chemical Society* **2007**, *129*, 13970-13977, 10.1021/ja074704l.
7. Arhel, N.; Genovesio, A.; Kim, K.A.; Miko, S.; Perret, E.; Olivo-Marin, J.C.; Shorte, S.; Charneau, P. Quantitative four-dimensional tracking of cytoplasmic and nuclear hiv-1 complexes. *Nat. Methods* **2006**, *3*, 817-824, 10.1038/nmeth931.
8. Endreß, T.; Lampe, M.; Briggs, J.A.G.; Kräusslich, H.-G.; Bräuchle, C.; Müller, B.; Lamb, D.C. Hiv-1–cellular interactions analyzed by single virus tracing. *European Biophysics Journal* **2008**, *37*, 1291-1301, 10.1007/s00249-008-0322-z.
9. Seisenberger, G.; Ried, M.U.; Endreß, T.; Büning, H.; Hallek, M.; Bräuchle, C. Real-time single-molecule imaging of the infection pathway of an adeno-associated virus. *Science* **2001**, *294*, 1929.
10. Bally, M.; Gunnarsson, A.; Svensson, L.; Larson, G.; Zhdanov, V.P.; Hook, F. Interaction of single viruslike particles with vesicles containing glycosphingolipids. *Physical Review Letters* **2011**, *107*, 188103, 10.1103/PhysRevLett.107.188103.
11. Peerboom, N.; Block, S.; Altgarde, N.; Wahlsten, O.; Moller, S.; Schnabelrauch, M.; Trybala, E.; Bergstrom, T.; Bally, M. Binding kinetics and lateral mobility of hsv-1 on end-grafted sulfated glycosaminoglycans. *Biophys. J.* **2017**, *113*, 1223-1234, 10.1016/j.bpj.2017.06.028.
12. Baumgärtel, V.; Ivanchenko, S.; Dupont, A.; Sergeev, M.; Wiseman, P.W.; Kräusslich, H.-G.; Bräuchle, C.; Müller, B.; Lamb, D.C. Live-cell visualization of dynamics of hiv budding site interactions with an escrt component. *Nature Cell Biology* **2011**, *13*, 469, 10.1038/ncb2215 https://www.nature.com/articles/ncb2215#supplementary-information.
13. Chojnacki, J.; Staudt, T.; Glass, B.; Bingen, P.; Engelhardt, J.; Anders, M.; Schneider, J.; Müller, B.; Hell, S.W.; Kräusslich, H.-G. Maturation-dependent hiv-1 surface protein redistribution revealed by fluorescence nanoscopy. *Science* **2012**, *338*, 524.
14. Jouvenet, N.; Simon, S.M.; Bieniasz, P.D. Imaging the interaction of hiv-1 genomes and gag during assembly of individual viral particles. *Proc. Natl. Acad. Sci. U. S. A.* **2009**, *106*, 19114-19119, 10.1073/pnas.0907364106.

15. Prescher, J.; Baumgärtel, V.; Ivanchenko, S.; Torrano, A.A.; Bräuchle, C.; Müller, B.; Lamb, D.C. Super-resolution imaging of escrt-proteins at hiv-1 assembly sites. *PLoS Pathog.* **2015**, *11*, e1004677, 10.1371/journal.ppat.1004677.
16. Sarkar, A.; Robertson, R.B.; Fernandez, J.M. Simultaneous atomic force microscope and fluorescence measurements of protein unfolding using a calibrated evanescent wave. *Proc. Natl. Acad. Sci. U. S. A.* **2004**, *101*, 12882-12886, 10.1073/pnas.0403534101.
17. Ewers, H.; Smith, A.E.; Sbalzarini, I.F.; Lilie, H.; Koumoutsakos, P.; Helenius, A. Single-particle tracking of murine polyoma virus-like particles on live cells and artificial membranes. *Proc. Natl. Acad. Sci. U. S. A.* **2005**, *102*, 15110-15115, 10.1073/pnas.0504407102.
18. Sardo, L.; Hatch, S.C.; Chen, J.B.; Nikolaitchik, O.; Burdick, R.C.; De, C.; Westlake, C.J.; Lockett, S.; Pathak, V.K.; Hu, W.S. Dynamics of hiv-1 rna near the plasma membrane during virus assembly. *J. Virol.* **2015**, *89*, 10832-10840, 10.1128/jvi.01146-15.
19. Costello, D.A.; Whittaker, G.R.; Daniel, S. Variations in ph sensitivity, acid stability, and fusogenicity of three influenza virus h3 subtypes. *J. Virol.* **2015**, *89*, 350-360, 10.1128/jvi.01927-14.
20. Brandenburg, B.; Lee, L.Y.; Lakadamyali, M.; Rust, M.J.; Zhuang, X.W.; Hogle, J.M. Imaging poliovirus entry in live cells. *PLoS. Biol.* **2007**, *5*, 1543-1555, 10.1371/journal.pbio.0050183.
21. Ivanchenko, S.; Godinez, W.J.; Lampe, M.; Kräusslich, H.-G.; Eils, R.; Rohr, K.; Bräuchle, C.; Müller, B.; Lamb, D.C. Dynamics of hiv-1 assembly and release. *PLoS Pathog.* **2009**, *5*, e1000652, 10.1371/journal.ppat.1000652.
22. Tokunaga, M.; Imamoto, N.; Sakata-Sogawa, K. Highly inclined thin illumination enables clear single-molecule imaging in cells. *Nat. Methods* **2008**, *5*, 159, 10.1038/nmeth1171 https://www.nature.com/articles/nmeth1171#supplementary-information.
23. Burdick, R.C.; Hu, W.-S.; Pathak, V.K. Nuclear import of apobec3f-labeled hiv-1 preintegration complexes. *Proceedings of the National Academy of Sciences* **2013**, *110*, E4780.
24. Chin, Christopher R.; Perreira, Jill M.; Savidis, G.; Portmann, Jocelyn M.; Aker, Aaron M.; Feeley, Eric M.; Smith, Miles C.; Brass, Abraham L. Direct visualization of hiv-1 replication intermediates shows that capsid and cpsf6 modulate hiv-1 intra-nuclear invasion and integration. *Cell Reports 13*, 1717-1731, 10.1016/j.celrep.2015.10.036.
25. Mamede, J.I.; Cianci, G.C.; Anderson, M.R.; Hope, T.J. Early cytoplasmic uncoating is associated with infectivity of hiv-1. *Proceedings of the National Academy of Sciences* **2017**, *114*, E7169.
26. Oreopoulos, J.; Berman, R.; Browne, M. Chapter 9 - spinning-disk confocal microscopy: Present technology and future trends. In *Methods in cell biology*, Waters, J.C.; Wittman, T., Eds. Academic Press: 2014; Vol. 123, pp 153-175, https://doi.org/10.1016/B978-0-12-420138-5.00009-4.
27. Ma, Y.; He, Z.; Tan, T.; Li, W.; Zhang, Z.; Song, S.; Zhang, X.; Hu, Q.; Zhou, P.; Wu, Y., *et al.* Real-time imaging of single hiv-1 disassembly with multicolor viral particles. *ACS Nano* **2016**, *10*, 6273-6282, 10.1021/acsnano.6b02462.
28. Kapanidis, A.N.; Lee, N.K.; Laurence, T.A.; Doose, S.; Margeat, E.; Weiss, S. Fluorescence-aided molecule sorting: Analysis of structure and interactions by alternating-laser excitation of single molecules. *Proc. Natl. Acad. Sci. U. S. A.* **2004**, *101*, 8936-8941, 10.1073/pnas.0401690101.
29. Kapanidis, A.N.; Laurence, T.A.; Lee, N.K.; Margeat, E.; Kong, X.; Weiss, S. Alternating-laser excitation of single molecules. *Accounts of Chemical Research* **2005**, *38*, 523-533, 10.1021/ar0401348.
30. Muller, B.K.; Zaychikov, E.; Brauchle, C.; Lamb, D.C. Pulsed interleaved excitation. *Biophys. J.* **2005**, *89*, 3508-3522, 10.1529/biophysj.105.064766.

31. Becker, W.; Hickl, H.; Zander, C.; Drexhage, K.H.; Sauer, M.; Siebert, S.; Wolfrum, J. Time-resolved detection and identification of single analyte molecules in microcapillaries by time-correlated single-photon counting (tcspc). *Rev. Sci. Instrum.* **1999**, *70*, 1835-1841, 10.1063/1.1149677.
32. Hendrix, J.; Lamb, D.C. Implementation and application of pulsed interleaved excitation for dual-color fcs and rics. In *Fluorescence spectroscopy and microscopy: Methods and protocols*, Engelborghs, Y.; Visser, A.J.W.G., Eds. Humana Press: Totowa, NJ, 2014; pp 653-682, 10.1007/978-1-62703-649-8_30.
33. Hendrix, J.; Schrimpf, W.; Holler, M.; Lamb, D.C. Pulsed interleaved excitation fluctuation imaging. *Biophys. J.* **2013**, *105*, 848-861, 10.1016/j.bpj.2013.05.059.
34. Kudryavtsev, V.; Sikor, M.; Kalinin, S.; Mokranjac, D.; Seidel, C.A.M.; Lamb, D.C. Combining mfd and pie for accurate single-pair forster resonance energy transfer measurements. *ChemPhysChem* **2012**, *13*, 1060-1078, 10.1002/cphc.201100822.
35. Berezin, M.Y.; Achilefu, S. Fluorescence lifetime measurements and biological imaging. *Chemical reviews* **2010**, *110*, 2641-2684, 10.1021/cr900343z.
36. Sun, Y.; Hays, N.M.; Periasamy, A.; Davidson, M.W.; Day, R.N. Monitoring protein interactions in living cells with fluorescence lifetime imaging microscopy. *Methods in enzymology* **2012**, *504*, 371-391, 10.1016/B978-0-12-391857-4.00019-7.
37. Becker, W. Fluorescence lifetime imaging – techniques and applications. *Journal of Microscopy* **2012**, *247*, 119-136, 10.1111/j.1365-2818.2012.03618.x.
38. Suhling, K.; Hirvonen, L.M.; Levitt, J.A.; Chung, P.-H.; Tregidgo, C.; Le Marois, A.; Rusakov, D.A.; Zheng, K.; Ameer-Beg, S.; Poland, S., *et al.* Fluorescence lifetime imaging (flim): Basic concepts and some recent developments. *Medical Photonics* **2015**, *27*, 3-40, https://doi.org/10.1016/j.medpho.2014.12.001.
39. Ghosh, S.; Saha, S.; Goswami, D.; Bilgrami, S.; Mayor, S. Dynamic imaging of homo-fret in live cells by fluorescence anisotropy microscopy. In *Methods in enzymology, vol 505: Imaging and spectroscopic analysis of living cells: Live cell imaging of cellular elements and functions*, Conn, P.M., Ed. Elsevier Academic Press Inc: San Diego, 2012; Vol. 505, pp 291-327, 10.1016/b978-0-12-388448-0.00024-3.
40. Weidtkamp-Peters, S.; Felekyan, S.; Bleckmann, A.; Simon, R.; Becker, W.; Kuhnemuth, R.; Seidel, C.A.M. Multiparameter fluorescence image spectroscopy to study molecular interactions. *Photochemical & Photobiological Sciences* **2009**, *8*, 470-480, 10.1039/B903245M.
